# Comorbidity Between Math and Reading Problems: Is Phonological Processing a Mutual Factor?

**DOI:** 10.3389/fnhum.2020.577304

**Published:** 2021-01-07

**Authors:** Tonje Amland, Arne Lervåg, Monica Melby-Lervåg

**Affiliations:** ^1^Department of Special Needs Education, University of Oslo, Oslo, Norway; ^2^Department of Education, University of Oslo, Oslo, Norway

**Keywords:** reading, math, numeracy, comorbidity, phonological awareness

## Abstract

There is a relationship between reading and math skills, as well as comorbidity between reading and math disorders. A mutual foundation for this comorbidity could be that the quality of phonological representations is important for both early reading and arithmetic. In this study, we examine this hypothesis in a sample traced longitudinally from preschool to first grade (*N* = 259). The results show that phonological awareness does not explain development in arithmetic, but that there is an indirect effect between phoneme awareness in preschool and arithmetic in first grade *via* phoneme awareness in first grade. This effect is, however, weak and restricted to verbal arithmetic and not arithmetic fluency. This finding is only partly in line with other studies, and a reason could be that this study more strongly controls for confounders and previous skills than other studies.

## Introduction

Mastering reading and math is vital for not only academic performance but also important life skills critical for employment and participation in society. Researchers have long known that there is a rather close relationship between reading and mathematics. The two skills correlate moderately to highly (Peng et al., 2020), and a large number of children with dyslexia also struggle with math difficulties. Reversely, many children with dyscalculia also have difficulties in reading (Joyner and Wagner, 2020). A question yet to be answered is what kind of foundational skills might be a common factor underlying reading and arithmetic. One hypothesis that has gained support is that phonological processing underlies not only early reading skills but also foundational mathematic skills. In this study, we investigate this hypothetical cause of the relationship between early reading and mathematics skills and thus focus on the relationship between math and early reading.

## To What Extent Are Reading and Arithmetic Correlated?

Although correlation does not imply causation, a correlation is often a starting point for disentangling causality. Studies have shown that reading and mathematics are correlated both at the genetic level and in brain activation patterns (Shaywitz et al., 1998; Temple et al., 2001; Ashkenazi et al., 2013; Pollack and Ashby, 2018). These correlations are also reflected at a behavioral level: on a broader level, a recent meta-analysis illustrated that the mean correlation between language and math across 392 independent samples was moderate (*r* = 0.42; Peng et al., 2020). This correlation, unsurprisingly, is also reflected in children at the lower end of the distribution with math and reading problems. A recent review showed that children with a math disorder were over two times more likely to also have a reading disorder than those without a math disorder (odds ratio = 2.12; Wagner et al., 2019). This result coincides with findings from other meta-analyses (see Swanson et al., 2009; Landerl and Moll, 2010).

## Phonological Processing as A Foundation for Arithmetic as Well as Word Decoding

Theoretically, the “triple code model,” the most influential framework for understanding early number processing, assumes a pathway from language to numeracy (Dehaene, 1992). An important question then is which aspects of language might be causally related to mathematics. One candidate is skills related to phonology. The consensus concerning reading is that phonological processing of sounds in words (often measured with phoneme awareness tasks) is an important causal precursor of growth in word decoding and the primary cause of reading problems such as dyslexia (Melby-Lervåg et al., 2012; Caravolas et al., 2013; Snowling and Melby-Lervåg, 2016). In a meta-analytic comparison of the effectiveness of reading interventions, phoneme awareness programs were found to be among the most successful approaches to boosting children’s reading skills over time (Suggate, 2016). To master such phonological awareness tasks, a child must be able to encode, maintain, and reproduce accurate representations of words from memory. Performance on phonological awareness tests is therefore considered to reflect the quality of phonological representations stored in memory. Thus, the more fine-grained and detailed the phonological processing and representations are, the better the performance on phonological awareness tests.

However, performance on such phonological processing tasks is highly correlated with not only decoding but also early mathematics skills. In particular, performance on such phonological processing tasks is strongly correlated with arithmetic, the part of mathematics that concerns numbers and basic operations on them—addition, subtraction, multiplication, and division. A review by Peng et al. (2020) showed an average moderate correlation between phonological awareness and arithmetic (*r* = 0.35).

The quality of phonological representations can be important for arithmetic problem-solving in several ways. First, to solve a computation problem, a child must first transform the numbers and operators in the problem into a speech-based code (Dehaene, 1992; Hecht et al., 2001). Studies have shown that this Arabic-to-verbal translation appears to be routinely used by children not only to solve simple arithmetic problems but also for more general math computation problems, such as long division and fractions. A second stage during which phonological representations might be important is after the Arabic-to-verbal translation. The child must then process the phonological information using a specific task-solving strategy. For a simple arithmetic problem (4 + 3 = ?), one must retrieve the answer directly from long-term memory, and so the ability to solve such a problem is dependent on the storage of phonological information. Another alternative can be to use a counting-based strategy to retrieve the answer. The phonological system is then employed when the child uses the phonological codes for the number names in counting. Thus, there are several ways in which phonological processing may also yield a causal influence on numeracy. Phonological processing tasks are likely important for both decoding and arithmetic since both tasks depend on mental processes that use sound-based representations.

As for previous studies, some observational investigations have offered support for this theory. One study followed children from second to fifth grade (*N* = 201; Hecht et al., 2001). The researchers found that phonological processing was uniquely associated with the development of early arithmetic skills. Importantly, phonological processing (as measured by digit span, rapid naming, and phonological awareness) almost entirely accounted for the relationship between reading and math. However, the authors found no unique influence of phonological processing on arithmetic fluency speed from fourth to fifth grade. There are several potential explanations for this result. For example, the children could have been too old for phonological processing to have an influence. Another explanation might be that phonological processing (or, more specifically, the quality of phonological representations) influences accuracy but not speed. A study by De Smedt et al. (2010) of fourth- and fifth-grade children (*N* = 37) yielded results in line with those of Hecht et al. (2001): they found that the quality of phonological representations was particularly important for the retrieval of existing arithmetic facts (e.g., 2 + *3* = 5). Such single digits problems are easier and faster to retrieve compared to larger problems requiring procedural strategies.

Recently, a larger 1-year longitudinal study of Finnish 7-year-olds (*N* = 200) followed up on Hecht et al. (2001) and examined predictors of the covariance between reading and arithmetic fluency (Koponen et al., 2019). The results showed that phonological awareness is a unique longitudinal predictor of this variation. Along the same lines, a 1-year study of students in kindergarten and first grade by Cirino et al. (2018) examined predictors for the mutual variance between reading and math (*N* = 193). This study found that phonological awareness, together with other linguistic naming tasks, accounted for nearly the same amount of overlap as all predictors together. This finding matches that of an earlier concurrent study (*N* = 233) from the same research environment, which showed a relationship between both timed and untimed arithmetic tasks (Child et al., 2019).

Furthermore, a recent concurrent study of 5-year-old kindergarteners (*N* = 188) also suggests that phonological awareness is related to early arithmetic (Vanbinst et al., 2020). The main finding was that phonological awareness was related not only to early reading but also to early arithmetic. The relationship notably remained after controlling for early reading- and arithmetic-specific cognitive correlates. Finally, Singer et al. (2019) also found, using concurrent data on children aged 9–11 years (*N* = 262), that phonological awareness was uniquely related to simple arithmetic fluency computations.

Still, other observational studies have found no unique relationship between phonological awareness and arithmetic, despite high correlations between the two. Purpura et al. (2011) found that letter knowledge—but not phonological processing—uniquely predicted development in arithmetic from 1 year to the next. Moll et al. (2015) reported that children with arithmetic difficulties only, and not comorbid reading problems, had difficulties restricted only to the arithmetic domain, while children with only reading difficulties also had problems with tasks related to arithmetic exercises that involved a verbal code. The comorbid math/language group exhibited difficulties with both. Thus, in this study, only the result of the comorbid group was in line with the hypothesis that children with arithmetic difficulties have phonological problems. In a previous study by Landerl et al. (2009), findings suggested that dyslexia and dyscalculia have separable cognitive profiles; when comparing groups of dyslexic, dyscalculic, and dyslexic/dyscalculic children, the researchers found a phonological deficit in both dyslexic groups, but not in the dyscalculia-only group. Contrastingly, they found difficulties in symbolic and non-symbolic number processing in both groups with dyscalculic children, but not in the dyslexia-only group.

Thus, taken together, findings on the role of phonological processes as a foundation of arithmetic as well as word decoding are inconclusive. Two possible reasons for discrepancies in the findings are differences in age of the children studied as well as differences in tasks used to measure such phonological processes. It is possible that detection of this relationship depends on the degree of automated fact retrieval and whether or not the task used to measure phonological skill is a phonological awareness task or a different measure aimed at capturing other related processes. Phonological skills have been measured by different tasks reflecting different components, such as rapid automatized naming (RAN) and verbal working memory (Wagner and Torgesen, 1987; Hecht et al., 2001). However, results concerning rapid naming and phonology tasks tapping verbal working/short term memory can be difficult to interpret since it is not entirely clear what these components measure. Because the construct phonological processing has evolved over time, and recent theory relating to arithmetic has focused on phonological awareness, this is also the aspect of phonological processing assessed in the present study. Beyond phonological processes, it is also possible that other domain-general abilities can account for the observed relationship, as previous studies vary in the extent to which they include broader abilities.

## The Correlation Between Phonological Awareness and Arithmetic: Is It Spurious and Caused by Underlying General Cognitive Skills?

The first potential explanation for the correlation between phonological awareness and arithmetic is that general cognitive ability is the third variable that underlies both. Possible cognitive candidates that have been suggested to underlie this correlation are nonverbal abilities (Bull and Scerif, 2001; Geary, 2004) and general language skills (Moll et al., 2015). If this is correct, controlling for cognitive abilities in the relationship between phonological awareness and arithmetic would weaken the correlation or make it disappear entirely. A recent review by Peng et al. (2020) demonstrates that general intelligence explains a large proportion of the variance in the relationship between mathematics and language.

However, previous studies of phonological awareness and arithmetic have varied with the extent to which they take such variables into account: Hecht et al. (2001) controlled for general language skills and found that the relationship between phonological processing and arithmetic held. They did not, however, control for nonverbal abilities. Koponen et al. (2019) controlled for various memory-related variables but not verbal and nonverbal abilities, while Cirino et al. (2018) controlled for processing speed, visuospatial working memory, and nonverbal abilities. As for the concurrent studies, Child et al. (2019) controlled for working memory and processing speed; Vanbinst et al. (2020) controlled for nonverbal abilities; and Singer et al. (2019) controlled for both verbal and nonverbal abilities. Regarding those studies that did not find a relationship between phonological awareness and arithmetic, Purpura et al. (2011) controlled for nonverbal intelligence, while Moll et al. (2015) controlled for both verbal and nonverbal abilities.

## Phonological Awareness—More Important for Some Aspects of Arithmetic than Others?

As mentioned, the quality of the phonological representations can be important for arithmetic problem-solving in several ways. This also implies that the relationship can stronger for some types of computation than others. However, only one previous study has examined whether there are differential effects for different types of arithmetic types. This study found a relationship between phonological awareness and addition/subtraction fluency, but not with number knowledge or word problems (Singer et al., 2019). This distinction is potentially significant because it implies that the general language ability required for word problem-solving is separate from the phonological processes involved in efficient arithmetic fact retrieval.

The remaining studies have used a variety of tasks: Hecht et al. (2001) used a latent variable with a range of tasks such as untimed simple digit addition and subtraction, multi-digit addition, multiplication, division, fractions, and algebra as well as addition and subtraction fluency. Koponen et al. (2019) used a latent variable with addition and subtraction fluency and basic arithmetic, while Vanbinst et al. (2020) measured basic addition and subtraction accuracy. While such tasks are likely to trigger arithmetic fact retrieval due to small problem sizes and frequent administration with a time limit, they are rarely compared to other types of arithmetic tasks that may or may not also rely on phonological processes. Comparing additional types of arithmetic concerning their ties to phonological skill could provide clarity on which aspects of early number ability account for the observed relationships. In addition to procedural vs. fact-retrieval demands, task characteristics such as the degree of vocalization, working speed, and use of digits vs. number words might matter in terms of whether a relationship is detected.

## The Current Study

Taken together, several studies have pointed to phonological awareness as a candidate for explaining why there is a relationship between early reading and arithmetic. However, previous results have been inconclusive. Moreover, the studies have varied in whether they have controlled for confounders in their outcome measures. Furthermore, some of these studies have included tasks in their phonology construct tasks that would now be considered rather unconventional, such as rapid naming and verbal short-term memory measures. In the present study, we deal with these issues and examine the relationship between phonological awareness (measured with widely used task formats) and arithmetic in a large sample, controlling for measurement error and verbal and nonverbal abilities. We examine the following research questions:

• Can phoneme awareness in preschool predict both reading and arithmetic skills in first grade when controlling for early reading and number skills, general language skills, and nonverbal abilities?
• Does phoneme awareness predict reading and arithmetic concurrently, and are there indirect effects of phoneme awareness in preschool on arithmetic and reading outcomes through phoneme awareness in first grade?
• Does phoneme awareness relate differently to verbal arithmetic and arithmetic fluency?

## Materials and Methods

### Participants

We recruited a cohort of 259 Norwegian-speaking children (135 boys and 126 girls) with a mean age of 5.5 years (range 4.9–6.1 years, *SD* = 0.3) when the study began. The children were recruited from a district in southeastern Norway that compared to the national average on aspects relating to educational level and socioeconomic status (Statistics Norway, 2020a,b). Children diagnosed with severe learning or developmental disorders, such as autism, sensory impairments, or an intellectual disability, were excluded from the study during data collection.

We obtained informed consent for the children’s participation in the study from their parents. Additionally, the children gave verbal consent at each time point of the data collection.

At the first time point, the children were in their final year of preschool. Norwegian children enter primary school and begin formal literacy and numeracy instruction the year they turn six. No formal instruction in reading or math is given in preschool before children enter first grade in primary school. At the time of the testing in grade one, the children in our sample had had about 6 months of formal instruction, with some children yet to master reading and calculations. However, due to the early assessment point, there might be lower-performing children in our sample who will receive diagnoses as they age.

### Design and Procedure

The children were assessed individually on a range of measures of numeracy, literacy, and general cognition with a 12-month time interval in their respective kindergartens and later schools. The tests used in the data collection are mainly internationally established and widely used measures that have been adapted to Norwegian. See Table 1 for the descriptive statistics and reliabilities for all measures.

The tests were administrated individually in a fixed order by trained research assistants. The research assistants visited the children’s kindergartens and later schools three times in the weeks between early January and late March 2 years in a row. The tests were part of a larger test battery involving a range of cognitive abilities, and each of the three sessions lasted about 35–55 min, depending on the child’s working speed and needs.

**Table 1:** Descriptive statistics and reliability for all observed variables.

Task	*N*	Min-max	*M*	*SD*	Reliability ω
Preschool, 5 years					
Vocabulary (BPVS)	252	0–99	62.24	13.99	0.95
Grammar (TROG)	254	1–75	44.80	17.94	0.97
Ravens CPM	245	7–35	17.75	4.48	0.75
Matrices WPPSI	250	1–22	11.62	5.32	0.92
Number naming	254	1–13	8.32	2.60	0.82
Verbal arithmetic, addition	254	0–11	5.59	3.81	0.92
Letter knowledge, vowels	254	0–9	4.87	2.67	0.82^1^
Letter knowledge, consonants	254	0–17	9.33	6.07	0.82^1^
Phoneme isolation	254	2–24	11.31	6.55	0.95
First grade, 6 years					
Number naming	244	6–22	14.97	4.68	0.91
Number identification	244	1–8	5.44	1.77	0.71
Phoneme deletion, words	243	0–12	6.40	3.29	0.84
Phoneme deletion, non-words	243	0–12	4.72	3.38	0.85
Arithmetic fluency, addition	244	1–23	9.95	3.66	0.88
Arithmetic fluency, subtraction	244	0–20	6.08	3.87	0.89
Verbal arithmetic, addition	244	0–22	15.47	4.19	0.86
Verbal arithmetic, subtraction	244	0–14	9.82	4.25	0.93
Word reading A	252	0–83	21.72	14.02	0.94^1^
Word reading B	252	0–91	18.88	14.49	0.94^1^

*Note: ^1^Correlation between the two alternate forms*.

### Measures

The following indicators were used.

#### Preschool, Age Five

General language skills were measured with two indicators: first, receptive grammar skills were assessed using the Norwegian version of the Test for Reception of Grammar (TROG; version 2; Bishop, 2009). The Norwegian version of the TROG has been standardized and normed with a Norwegian sample (Lyster and Horn, 2009). In this task, the child hears sentences of increasing complexity and selects a picture for each sentence. Example sentences include the following: “The girl is sitting on the table” and “The blue cup is on top of the small yellow book.” The test contains 80 four-choice items and is discontinued when the child makes one or more errors in five consecutive blocks.

Second, receptive vocabulary skills were assessed with a translated version of the British Picture Vocabulary Scale (BPVS; Dunn and Dunn, 2009). The Norwegian version of the BPVS has also been standardized and normed with a Norwegian sample (Lyster et al., 2010). The test requires the child to match a spoken word with one of four presented pictures. This instrument has a total of 144 items of increasing difficulty, and test administration ends when the child makes eight or more errors in a block of 12 items.

Nonverbal general abilities were also measured with two indicators: first, Raven’s Coloured Progressive Matrices (CPM) is a test designed for children aged 5–11 (Raven, 2000). The instrument measures nonverbal intelligence and abstract reasoning abilities, and very limited verbal instruction is given by the test administrator. The child sees a set of illustrations with a piece missing and is then required to identify the construction pattern of increasingly complex geometrical figures by selecting the piece required to complete the set. There are 36 items organized in sets, and the test is administered without time constraints.

Second, the matrix reasoning task from the Wechsler Preschool and Primary Scale of Intelligence, 4th edition (WPPSI-IV) is also an index of nonverbal abilities (Wechsler, 2012). In this task, the child sees an incomplete matrix and is required to select the missing alternative that completes the matrix. Picture or figure analogies must be nonverbally identified for the child to select the correct response. An example of such a task is a matrix with a horse, a barn, and a fish, as well as an empty square. Among the alternatives for the missing piece is an empty fishbowl, which would be the correct choice. As the child progresses, items become gradually more abstract and complex.

Number word knowledge in preschool was measured with a number-naming task. The child was shown a series of printed numbers and asked to name them: “Which number is this?” The numbers increased in size and range from single- to four-digit numbers.

Verbal arithmetic skills in preschool were measured with a verbal addition task. The task consisted of linguistically simple addition problems presented orally and calculated mentally (e.g., “What is seven plus nine?”). The child responded verbally. For 5-year-old children, we only used addition as an indicator of arithmetic skills due to the large number of children who are unfamiliar with subtraction at this stage.

Letter knowledge in preschool was assessed by having the child give the name or sound of letters in the Norwegian alphabet shown on a sheet of paper, ordered alphabetically. In task one, the printed letters were vowels (e.g., A, E, I …) and in task two, the letters were consonants (B, D, F …).

Phoneme awareness at age five was measured with a phoneme isolation task. The child was asked to identify a phoneme in a word read aloud by the test administrator (e.g., “What is the first sound in boat?”). We used two blocks where the child was asked to identify the first sound of a given word in block one and the last sound of the word in block two.

The four first items in each block were accompanied by pictures for support. The items consisted of three or four-letter words [either consonant-vowel-consonant (CVC), CCVC, or CVCC].

### First Grade, Age Six

Number word knowledge in first grade was measured by two tasks assessing the child’s knowledge of the correspondence between digits and number words.

Number naming. The first number-knowledge task was the same as that used in the previous year but extended to include more difficult items (i.e., larger numbers with up to four digits). The child was shown a series of printed numbers and asked to name them (“Which number is this?”).

Number identification. The children were also given a number identification task, similar to the task used by Göbel et al. (2014). Number identification entailed drawing a circle around one of five printed numbers for each number read aloud by the test administrator. For example, the test administrator might read “163”, and the child would then mark one of five options presented on a sheet (136, 10,063, 13, 163, 16). The target numbers ranged from one to three digits.

Phoneme awareness was assessed as follows: two phoneme deletion tasks were administered, one with words and one with non-words. The tasks required the child to delete sounds in the beginning, middle, or end of the words. A translated example is “Say ‘cat’ without saying ‘/k/’.”

Arithmetic fluency was measured with two tasks, one with addition problems and one with subtraction problems. The subtasks were taken from the Test of Basic Arithmetic and Numeracy Skills (TOBANS; Brigstocke et al., 2016). The child completed three practice items for each set to ensure that they understood the mathematical operation required and was then asked to solve as many problems as possible with pencil and paper within 60 s. The number of correct answers was recorded.

Verbal arithmetic skills were assessed in first grade with linguistically simple arithmetic problems presented orally and calculated mentally. The test had two parts; part one consisted of increasingly difficult addition problems, and part two contained subtraction problems. The child responded verbally and the number of correct responses was recorded.

Reading efficiency was assessed by requiring children to read aloud two lists of words from a Norwegian translation of the Test of Word Reading Efficacy (TOWRE; Torgesen et al., 1999). Children are given 45 s to read aloud as many words as they can from each list. The score is the number of items read and pronounced correctly.

## Results

### Descriptive Statistics and Correlations

Table 1 shows the means, standard deviations, minimum and maximum scores, and reliabilities for all measures, and the correlations between all observed variables are available in online supplement (Supplementary Table 1). Table 1 shows that all variables had decent reliability. All further analyses were done in Mplus version 8 (Muthén and Muthén, 1998-2017) using the full information maximum likelihood approach to handle missing values.

### Confirmatory Factor Analyses

To test the relations between the latent constructs and their respective observed indicators (i.e., the measurement model) and between the latent constructs themselves, we estimated a confirmatory factor analysis (CFA) in which we included all variables. Six constructs were measured in preschool (nonverbal abilities, language, letter knowledge, phoneme awareness, number knowledge, and verbal arithmetic) and five constructs were measured in first grade (phoneme awareness, number knowledge, verbal arithmetic, arithmetic fluency, and word reading). See Table 2 for the indicators of the constructs. This model had an excellent fit to the data, ${\text{χ}}_{\left(\text{99}\right)}^{\text{2}}$ = 109.902, *p* = 213; RMSEA = 0.021 (90% CI = 0.000, 0.040); TLI = 0.993; SRMR = 0.026. Table 2 shows the factor loadings and factor correlations. As illustrated, the factor loadings were relatively strong for most observed indicators, except matrixes (*λ* = 0.407). Regarding the hypotheses, strong correlations were found between phoneme awareness and both reading and verbal arithmetic. The correlations between phoneme awareness and arithmetic fluency were somewhat lower.

**Table 2:** Standardized factor loadings for all latent constructs and their respective indicators plus factor correlations between the latent constructs.

Latent constructs and observed indicators	Factor loadings^1^	1	2	3	4	5	6	7	8	9	10	11
1_Nonverbal abilities PS		1	0.456	0.359	0.392	0.353	0.348	0.200^3^	0.426	0.306	0.289	0.183^3^
Raven CPM	0.953											
Matrices WPPSI	0.407											
2_Language_PS			1	0.387	0.390	0.266	0.511	0.360	0.535	0.366	0.336	0.334
Vocabulary (BPVS)	0.531											
Grammar (TROG)	0.871											
3_Letter knowledge PS				1	0.731	0.408	0.676	0.503	0.549	0.370	0.304	0.577
Consonants	0.908											
Vowels	0.904											
4_Number knowledge PS					1	0.708	0.490	0.457	0.538	0.495	0.558	0.586
Number naming PS	0.905^2^											
5_Number knowledge G1						1	0.318	0.446	0.524	0.686	0.672	0.469
Number naming	0.845											
Number identification	0.825											
6_Phoneme awareness PS							1	0.508	0.538	0.382	0.246	0.540
Phoneme isolation	0.964^2^											
7_Phoneme awareness G1								1	0.540	0.643	0.384	0.638
Phoneme deletion W	0.849											
Phoneme deletion NW	0.906											
8_Verbal arithmetic PS									1	0.593	0.490	0.428
Addition	0.928^2^											
9_Verbal arithmetic G1										1	0.702	0.443
Verbal addition	0.860											
Verbal subtraction	0.581											
10_Arithmetic fluency G1											1	0.432
Addition fluency	0.848											
Subtraction fluency	0.796											
11_Reading												1
Word reading A	0.992											
Word reading B	0.948											

*Note: ^1^All factor loading have p-values <0.001. ^2^The residual of the observed indicator is fixed to reflect the reliability of the variable. ^3^The p-value of the correlation is <0.05. Correlations without superscript have p-values <0.001*.

### Does Phoneme Awareness Predict Reading as Well as Verbal Arithmetic and Arithmetic Fluency?

To test this, we first estimated a structural equation model (SEM) in which we regressed reading, verbal arithmetic, and arithmetic fluency in first grade on phoneme awareness in preschool together with the control constructs letter knowledge, number-word knowledge, verbal arithmetic, language, and nonverbal abilities in preschool. Also, phoneme awareness, letter knowledge, number-word knowledge, and verbal arithmetic skills in preschool were regressed on language and nonverbal abilities in preschool.

**Figure 1:**
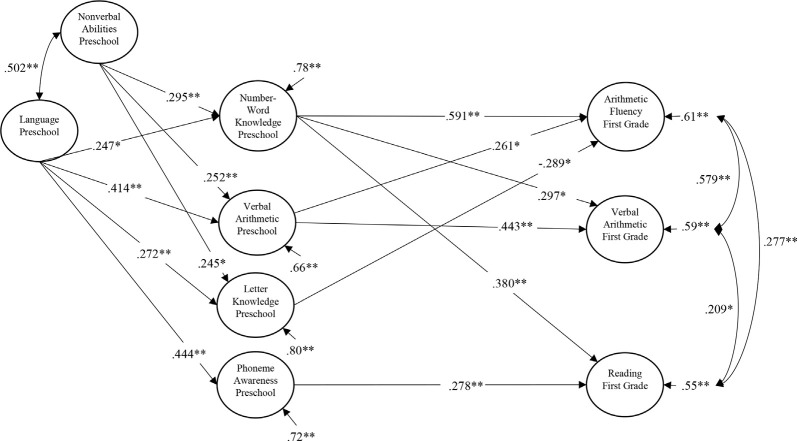
Structural equation path model showing the longitudinal relations between Time 1 preschool preliteracy and numeracy skills, and Time 2 first grade reading and arithmetic. Single-headed arrows between the latent variables are standardized regression weights. Double-headed arrows indicate correlations (covariances). *p < 0.05, **p <0.01.

As shown in Figure 1, phoneme awareness in preschool predicted reading but not verbal arithmetic and arithmetic fluency in first grade. Number-word knowledge in preschool predicted both reading and the two arithmetic constructs in first grade; verbal arithmetic in preschool predicted both verbal arithmetic and arithmetic fluency in first grade. Also, there was a negative suppression effect of letter knowledge in preschool on arithmetic fluency in first grade. Language in preschool directly predicted phoneme awareness, letter knowledge, number-word knowledge, and verbal arithmetic in preschool and indirectly predicted reading and the two arithmetic constructs in first grade. The same was true for nonverbal abilities in preschool, with the exception that this construct did not predict phoneme awareness in preschool beyond language. This model had an excellent fit to the data, ${\text{χ}}_{\left(\text{56}\right)}^{\text{2}}$ = 59.392, *p* = 353; RMSEA = 0.015 (90% CI = 0.000, 0.042); TLI = 0.997; SRMR = 0.023.

**Figure 2:**
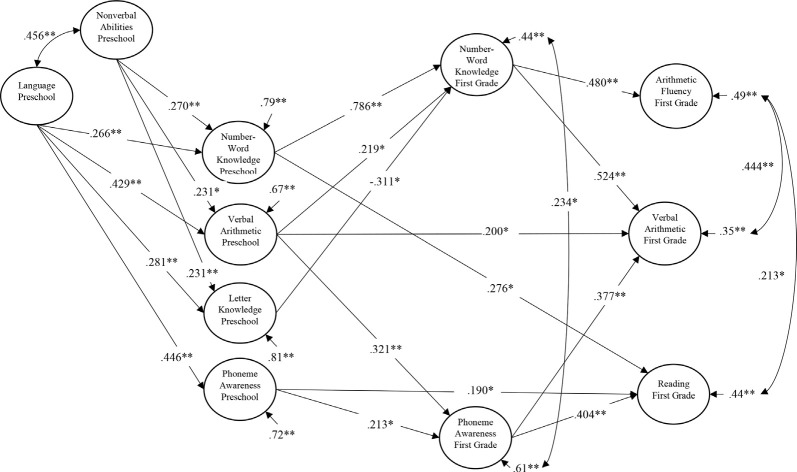
Structural equation path model showing the longitudinal relations between Time 1 preschool preliteracy and numeracy skills, and Time 2 first grade phoneme awareness, number word knowledge, reading, and arithmetic. Single-headed arrows between the latent variables are standardized regression weights. Double-headed arrows indicate correlations (covariances). *p < 0.05, **p <0.01.

Next, we added phoneme awareness and number naming in first grade as additional predictors of reading, verbal arithmetic, and arithmetic fluency in first grade. This was done to see if phoneme awareness predicted reading, verbal arithmetic, and arithmetic fluency concurrently, and if there were indirect effects of phoneme awareness on these three outcomes through phoneme awareness in first grade. As shown in Figure 2, and partially in line with the phonological hypothesis, phoneme awareness in first grade did predict both reading and verbal arithmetic but not arithmetic fluency in first grade. In addition to phoneme awareness in first grade, both phoneme awareness and number-word knowledge in preschool predicted reading in first grade, and verbal arithmetic and number-word knowledge in preschool predicted verbal arithmetic in first grade. Only number-word knowledge in first grade predicted arithmetic fluency in first grade beyond all other potential predictors. The indirect effect of phoneme awareness in preschool on reading and verbal arithmetic was 0.086 (bootstrapped 95% CI = 0.001, 0.191) and 0.080 (bootstrapped 95% CI = 0.001, 0.173), respectively. However, it should also be noted that phoneme awareness in preschool had a direct effect on reading so that the total effect of PA in preschool on reading in first grade was *β* = 0.279 (95% CI = 0.098, 0.429). This model had the same excellent fit to the data as the full CFA explained above.

In both these models shown in Figures 1, 2, there was an unexpected negative path from preschool letter knowledge to either first-grade arithmetic fluency (1) or first-grade number-word knowledge (Figure 2). These paths seemed to be caused by the high correlation between preschool letter knowledge and preschool number-word knowledge (*r* = 0.731). Deleting number-word knowledge from the model resulted in these two paths becoming positive but nonsignificant (*β* = 0.080, *p* = 0.462 for first grade arithmetic fluency in Figure 1 and *β* = 0.178, *p* = 0.078 for first grade number-word knowledge in Figure 2). It should however be mentioned that all of the eight latent predictor variables in Figure 2 were within the tolerances for commonly suggested thresholds for potential collinearity problems (e.g., all tolerances were above 0.2) and that the main results concerning the hypothesized relationships between phoneme awareness and arithmetic and reading did not change as a function of deleting the preschool number-word knowledge variable.

## Discussion

This study has revealed several interesting findings that add to the literature about the relationship between phonological awareness and arithmetic. First, we did not find that phoneme awareness can predict the development of arithmetic from preschool to first grade when early reading, arithmetic, general language skills, and nonverbal abilities are controlled for. However, we did find that phoneme awareness is concurrently related to arithmetic in first grade and that there is an indirect effect of phoneme awareness in preschool on arithmetic in first grade *via* phoneme awareness in first grade. Moreover, the relationship between phoneme awareness and arithmetic is restricted to verbal arithmetic—simple arithmetic problems presented orally and calculated mentally—and not arithmetic fluency.

### Phoneme Awareness in Preschool Does Not Predict the Development of Arithmetic Skills in First Grade

The finding that phoneme awareness in preschool does not predict the development of arithmetic skills in first grade when controlling for early reading and number skills, general language skills, and nonverbal abilities contrasts with the two other longitudinal studies in this area. Hecht et al. (2001) found a uniquely direct relationship between phonological awareness and the development of early arithmetic. However, that study did not control for nonverbal abilities, and the researchers employed a rather broad phonological processing construct that also integrated verbal short-term memory and rapid naming. Thus, these two factors can perhaps explain why they found a different result. Koponen et al. (2019) also reported that phonological awareness is a unique longitudinal predictor of the covariance between reading and arithmetic fluency. Similarly, they did not control for verbal or nonverbal intelligence, and this might explain the differences in our findings. Thus, our study overall employed stricter controls, in particular for previous skills, but also confounders such as verbal and nonverbal abilities. We also controlled for measurement error using latent variables. These factors together could explain why we found that phoneme awareness did not predict development, while other studies have come to the opposite conclusion.

### An Indirect Effect of Phoneme Awareness in Preschool on Arithmetic in First Grade

While we did not find a direct effect of phoneme awareness in preschool on arithmetic development, we did observe an indirect effect from phoneme awareness preschool *via* phoneme awareness in first grade. Notably, our study is the first to examine such indirect effects. As for the size of this effect, the indirect effect implies that being one standard deviation above the mean in phoneme awareness in preschool is associated with being 0.08 standard deviations above the mean in verbal arithmetic at age six. Thus, the effect is rather limited in size. Language-related interventions have been suggested as a tool for ameliorating math problems. For instance, for language comprehension, Fuchs et al. (2020) recently demonstrated the effects of a vocabulary and language intervention on word problem-solving. If we had found a large indirect effect, one could perhaps suggest something similar for phonology—that number-related phoneme awareness interventions in preschool could ameliorate arithmetic difficulties. However, since the effect size was rather limited, the prospects for training effects are also rather limited.

### Phoneme Awareness Is Related to Verbal Arithmetic but Not Arithmetic Fluency

Our study found that phoneme awareness was related to verbal arithmetic when children were asked to solve a single-digit addition or subtraction task without time limits, but not to single-digit arithmetic fluency tasks that were timed and answered on paper with no verbal response needed. No other studies have examined differential effects on arithmetic accuracy vs. fluency; the two previous longitudinal studies had both types of tasks mixed in a latent variable. Among the concurrent studies, one had fluency tasks and found a relationship (Singer et al., 2019), and the other had accuracy tasks and found a relationship (Vanbinst et al., 2020).

While efficient fact retrieval should be expected to contribute to success in both these task variations, there are some interesting differences in terms of the additional skill needed in each format. First, fluency tasks are timed and require faster fact retrieval and symbolic number-knowledge. In this process, Arabic-to-verbal translation—thought to evoke phonological processes—is likely an integral aspect. Second, the verbally presented math problems are connected to language in a broader sense than is the case with arithmetic fluency. In addition to calling for an accurate representation of number-word knowledge rather than Arabic digit knowledge, they might also evoke a greater degree of procedural strategies due to the working memory load since there is no permanent visual information. Third, the verbal arithmetic tasks were somewhat more complex, implying less fact retrieval and more procedural strategies for the more challenging items.

Thus, one reason that we found a relationship between verbal arithmetic and phoneme awareness could be that this task was presented orally to the children and that the children then used the phonological codes for a counting-based strategy to retrieve the answer. In contrast, the arithmetic fluency tasks were presented visually to the children. These children had just started to receive numeracy instruction and were unlikely to be able to use stored phonological information to retrieve the answers directly from long-term memory. Thus, they more likely used, for instance, strategies such as finger-counting or writing down dots to find the answers, and since the tasks were presented visually, the visual presentation format did perhaps not induce a lot of language processing. While confirmatory factor analyses indicated that these two constructs are best treated separately, the obvious similarities between the tasks call for further investigations of how distinguishable the two processes are over time and whether they evoke different arithmetic strategies.

As mentioned, the degree to which fact retrieval is established could determine the degree of reliance on phonological codes necessary for efficiency in the fluency tasks. Therefore, it cannot be ruled out that the outcome would have been different had the second assessment been carried out a year later.

## Conclusion and Prospects for Future Studies

This study revealed that phonological awareness does not explain development in arithmetic, but that there is an indirect effect between phoneme awareness in preschool and arithmetic in first grade *via* phoneme awareness in first grade. This effect is, however, weak and restricted to verbal arithmetic accuracy and not fluency.

To rule out third variables that might have caused the detection of a relationship between phoneme awareness and arithmetic in other studies, we controlled for general language and nonverbal abilities. Future studies should aim to also include a working memory component as working memory has been established as an important aspect of both phonological processing and early arithmetic (Peng et al., 2016).

We examined a sample of typically developing children and focused on correlations between continuous variables, and not on co-occurrences of categorical diagnoses. As pointed out by Krueger and Markon (2006), the term “comorbidity” could legitimately refer to either phenomenon. In light of this, an interesting question is whether these findings would have been replicated in a sample with children with reading disorders, arithmetic disorders, or both. As mentioned, Moll et al. (2015) found that only their comorbid group and the reading-disabled group had phonological awareness difficulties; those with only a math disorder did not. In general, both reading disorders and math disorders are defined using rather arbitrary cut-offs on continuously distributed variables. This perhaps explains these results, and predictors of individual differences may not be any different at the lower end of the distribution.

Furthermore, there is little evidence of a qualitative difference between those who have a learning disorder and those who are above the threshold (Snowling and Melby-Lervåg, 2016). A recent study adopted a transdiagnostic approach to examine whether brain differences relate to cognitive difficulties in childhood (Siugzdaite et al., 2020). The results indicated patterns suggesting that cognitive strengths and weaknesses cut across disorders and difficulties. According to Siugzdaite et al. (2020), “This stands in contrast to theories that specify a particular cognitive impairment as being *the* route to a particular diagnosed learning problem but is consistent with earlier ideas that developmental difficulties reflect complex patterns of associations rather than highly selective deficits” (p. 7). Thus, based on the continuous nature of reading and arithmetic, their correlation, and the limited support for qualitative differences between them, a similar pattern for children at the lower end of the distribution is perhaps likely. Future studies seeking to examine comorbidity might benefit from approaches where testable models can be used to identify the functional nature of disabilities and the need for intervention, as opposed to forming categories of atypical and typical development based on cut-off criteria (Branum-Martin et al., 2013).

## Data Availability Statement

The original contributions presented in the study are included in the article/Supplementary Materials, further inquiries can be directed to the corresponding author.

## Ethics Statement

The studies involving human participants were reviewed and approved by Norwegian centre for data research https://nsd.no/nsd/english/index.html. Written informed consent to participate in this study was provided by the participants’ legal guardian/next of kin.

## Author Contribuitons

All authors listed have made a substantial, direct and intellectual contribution to the work, and approved it for publication.

## Conflict of Interest

The authors declare that the research was conducted in the absence of any commercial or financial relationships that could be construed as a potential conflict of interest.
