# Supplementary material for: Comorbidity Between Math and Reading Problems: Is Phonological Processing a Mutual Factor?
Source: Front Hum Neurosci. 2021 Jan 7;14:577304. doi: 10.3389/fnhum.2020.577304 (PMC7817538; doi:10.3389/fnhum.2020.577304)
Supplement: Supplementary file 1 [file Table_3.docx]

Online supplement S1

| *Table 3: Correlations All Measures* | | | | | | | | | | | | | | | | | | |
| --- | --- | --- | --- | --- | --- | --- | --- | --- | --- | --- | --- | --- | --- | --- | --- | --- | --- | --- |
| Variable | **1** | **2** | **3** | **4** | **5** | **6** | **7** | **8** | **9** | **10** | **11** | **12** | **13** | **14** | **15** | **16** | **17** | **18** |
| *Preschool, 5 years* |  |  |  |  |  |  |  |  |  |  |  |  |  |  |  |  |  |  |
| Vocabulary BPVS | — |  |  |  |  |  |  |  |  |  |  |  |  |  |  |  |  |  |
| Grammar TROG | 0.46*** | — |  |  |  |  |  |  |  |  |  |  |  |  |  |  |  |  |
| Raven CPM | 0.24*** | 0.36*** | — |  |  |  |  |  |  |  |  |  |  |  |  |  |  |  |
| Matrices WPPSI | 0.17** | 0.26*** | 0.38*** | — |  |  |  |  |  |  |  |  |  |  |  |  |  |  |
| Number naming | 0.20** | 0.30*** | 0.34*** | 0.14* | — |  |  |  |  |  |  |  |  |  |  |  |  |  |
| Verbal addition | 0.26*** | 0.43*** | 0.37*** | 0.20** | 0.49*** | — |  |  |  |  |  |  |  |  |  |  |  |  |
| LK consonants | 0.27*** | 0.32*** | 0.29*** | 0.13* | 0.60*** | 0.47*** | — |  |  |  |  |  |  |  |  |  |  |  |
| LK vowels | 0.23*** | 0.26*** | 0.34*** | 0.11 | 0.60*** | 0.45*** | 0.82*** | — |  |  |  |  |  |  |  |  |  |  |
| Phoneme isolation | 0.27*** | 0.42*** | 0.32*** | 0.11 | 0.42*** | 0.48*** | 0.59*** | 0.59*** | — |  |  |  |  |  |  |  |  |  |
| *First grade, 6 years* |  |  |  |  |  |  |  |  |  |  |  |  |  |  |  |  |  |  |
| Number naming | 0.14* | 0.19** | 0.26*** | 0.00 | 0.56*** | 0.38*** | 0.30*** | 0.34*** | 0.27*** | — |  |  |  |  |  |  |  |  |
| Number ID | 0.15* | 0.16* | 0.31*** | 0.06 | 0.51*** | 0.42*** | 0.27*** | 0.29*** | 0.21** | 0.70*** | — |  |  |  |  |  |  |  |
| Phoneme del W | 0.22*** | 0.28*** | 0.16* | 0.16* | 0.36*** | 0.44*** | 0.39*** | 0.44*** | 0.42*** | 0.34*** | 0.30*** | — |  |  |  |  |  |  |
| Phoneme del NW | 0.19** | 0.23*** | 0.17** | 0.09 | 0.34*** | 0.40*** | 0.33*** | 0.38*** | 0.43*** | 0.38*** | 0.30*** | 0.77*** | — |  |  |  |  |  |
| Addition fluency | 0.10 | 0.24*** | 0.22*** | 0.09 | 0.42*** | 0.35*** | 0.16* | 0.24*** | 0.17** | 0.48*** | 0.46*** | 0.32*** | 0.26*** | — |  |  |  |  |
| Subtract fluency | 0.11 | 0.26*** | 0.22*** | 0.02 | 0.40*** | 0.39*** | 0.19** | 0.27*** | 0.20** | 0.43*** | 0.47*** | 0.26*** | 0.25*** | 0.68*** | — |  |  |  |
| Verbal addition | 0.12 | 0.25*** | 0.24*** | 0.11 | 0.38*** | 0.46*** | 0.27*** | 0.28*** | 0.28*** | 0.52*** | 0.49*** | 0.50*** | 0.44*** | 0.52*** | 0.47*** | — |  |  |
| Verbal subtraction | 0.16* | 0.24*** | 0.16* | 0.14* | 0.24*** | 0.33*** | 0.17** | 0.19** | 0.26*** | 0.28*** | 0.33*** | 0.35*** | 0.29*** | 0.37*** | 0.53*** | 0.49*** | — |  |
| Word reading A | 0.18** | 0.28*** | 0.18** | 0.15* | 0.52*** | 0.38*** | 0.53*** | 0.49*** | 0.51*** | 0.41*** | 0.37*** | 0.55*** | 0.55*** | 0.39*** | 0.36*** | 0.37*** | 0.27*** | — |
| Word reading B | 0.18** | 0.27*** | 0.15* | 0.15* | 0.49*** | 0.33*** | 0.48*** | 0.45*** | 0.50*** | 0.39*** | 0.34*** | 0.51*** | 0.52*** | 0.38*** | 0.36*** | 0.35*** | 0.26*** | 0.94*** |
| Note. * *p* < .05, ** *p* < .01, *** *p* < .001 | | | | | | | | | | | | | | | | | | |
